# ^1^H, ^13^C, ^15^N resonance assignment of human YAP 50–171 fragment

**DOI:** 10.1007/s12104-018-9805-8

**Published:** 2018-01-25

**Authors:** Michael Feichtinger, Tomáš Sára, Gerald Platzer, Borja Mateos, Fedir Bokhovchuk, Patrick Chène, Robert Konrat

**Affiliations:** 1 Max F. Perutz Laboratories, Department of Computational and Structural Biology, University of Vienna, Campus Vienna Biocenter 5, 1030 Vienna, Austria; 2 Disease Area Oncology, Novartis Institutes for Biomedical Research, 141 Klybeckstrasse, 4057 Basel, Switzerland

**Keywords:** YAP, Intrinsically disordered protein, Hippo pathway, NMR, TEAD

## Abstract

Yes associated protein (YAP) is an intrinsically disordered protein that plays a major role in the Hippo pathway, regulating organ size, cell proliferation, apoptosis, and is associated with cancer development. Therefore, the binding between YAP and TEAD is an interesting target for cancer therapy. The TEAD binding domain of YAP was mapped to protein residues 50–171. To obtain further structural insights into this 12 kDa segment of YAP, we report a backbone and a partial sidechain assignment of recombinant YAP 50–171.

## Biological context

Yes associated protein (YAP) is an partly intrinsically disordered protein transcribed into four different isoforms ranging from 326 to 504 residues (Santucci et al. 2015). YAP is a major target and a terminal effector of the Hippo pathway, important for the regulation of organ size (Zhao et al. 2011) and is associated with cancer development (Liu et al. 2012; Pobbati and Hong 2013; Ma et al. 2015; Pobbati et al. 2015). One of the functions of YAP is to bind to the TEAD transcription factors as a transcriptional coactivator. Therefore, the YAP:TEAD interaction might be a promising therapeutic approach in cancers where the Hippo pathway is deregulated. The formation of the YAP:TEAD complex is indirectly regulated by YAP phosphorylation, which prohibits nuclear translocation of YAP and leads to downregulation of genes related to proliferation; therefore, leading to apoptosis (Zhao et al. 2007).

Previously, the binding between YAP and TEAD was studied and the protein region 50–171 was identified as the TEAD binding domain (Vassilev et al. 2001) Furthermore, three binding interfaces were identified between YAP and TEAD (Li et al. 2010). Namely, a β-strand (residues 52–58), an α-helix (residues 61–73), and an Ω-loop (residues 86–100). Furthermore, key residues in the binding site and their contribution to the binding have been described (Mesrouze et al. 2017).

In order to gain further insights into the binding process and structural features of the bound and unbound state, we present a chemical shift assignment of YAP 50–171.

## Methods and experiments

### Sample preparation

The sequence coding for YAP residues 50–171 was amplified by PCR and cloned into a pETM14 vector by sequence and ligation independent cloning (SLIC) (Scholz et al. 2013). The plasmid, containing the fusion protein with a His6-tag at the N-terminus and a 3C protease cleavage site, was transformed into *Escherichia coli* Tuner™(DE3). Four liters of LB medium were inoculated with the overnight culture and incubated at 37 °C until an OD_600_ of 0.8. Cells were centrifuged and all pellets were combined and resuspended in one liter of M9 minimal medium containing 1 g/L ^15^N ammonium chloride (Sigma Aldrich) and 3 g/L ^13^C glucose (Cambridge Isotope) as the sole source of nitrogen and carbon, respectively (Marley et al. 2001). After an additional hour at 37 °C, expression was induced with the addition of 0.8 mM IPTG and cells were incubated for 18 h at 30 °C and consecutively harvested by centrifugation and stored at − 20 °C.

Frozen cell pellets were resuspended in lysis buffer (20 mM Tris, 150 mM NaCl, 30 mM Imidazole, pH 7.8), supplemented with 50 µL of Protease Inhibitor Cocktail (Thermo Scientific), and lysed by sonication on ice. Lysate was clarified by centrifugation and supernatant was loaded onto a HisTrap FF column and washed with 8 column volumes of wash buffer (20 mM Tris, 150 mM NaCl, 40 mM Imidazole, pH 7.8). After eluting with 15 ml of elution buffer (20 mM Tris, 150 mM NaCl, 300 mM Imidazole, 1 mM EDTA, pH 7.8), the eluate was concentrated to 2 ml and dialyzed overnight against an excess of NMR buffer (20 mM BisTris, 150 mM NaCl, 1 mM EDTA, pH 6.0). Consequently, His6-tag was removed by 3C protease cleavage overnight at 4 °C. NaCl was added to a final concentration of 1 M and the solution was heated to 90 °C for 10 min. After centrifugation, the supernatant was loaded onto a HiLoad 16/600 (GE Healthcare) size exclusion column equilibrated with NMR buffer. Purified protein, which was > 95% pure as assessed by SDS/PAGE, was concentrated to approximately 0.5 mM in NMR buffer containing 10% D_2_O as determined by Pierce BCA Protein Assay (Thermo Scientific).

### NMR spectroscopy

NMR experiments were performed at 298 K using a Bruker Avance III 800 MHz spectrometer. The backbone assignments were obtained using BEST-TROSY type versions of HNCANNH, HN(COCA)NNH, HNCACB, HN(CO)CACB, HNCO, and HN(CA)CO experiments (Solyom et al. 2013). Data was processed with NMRPipe (Delaglio et al. 1995) and spectra were assigned using CcpNmr (Vranken et al. 2005) and Sparky (Goddard and Kneller 2004).

## Assignments and data deposition

**Fig. 1:**
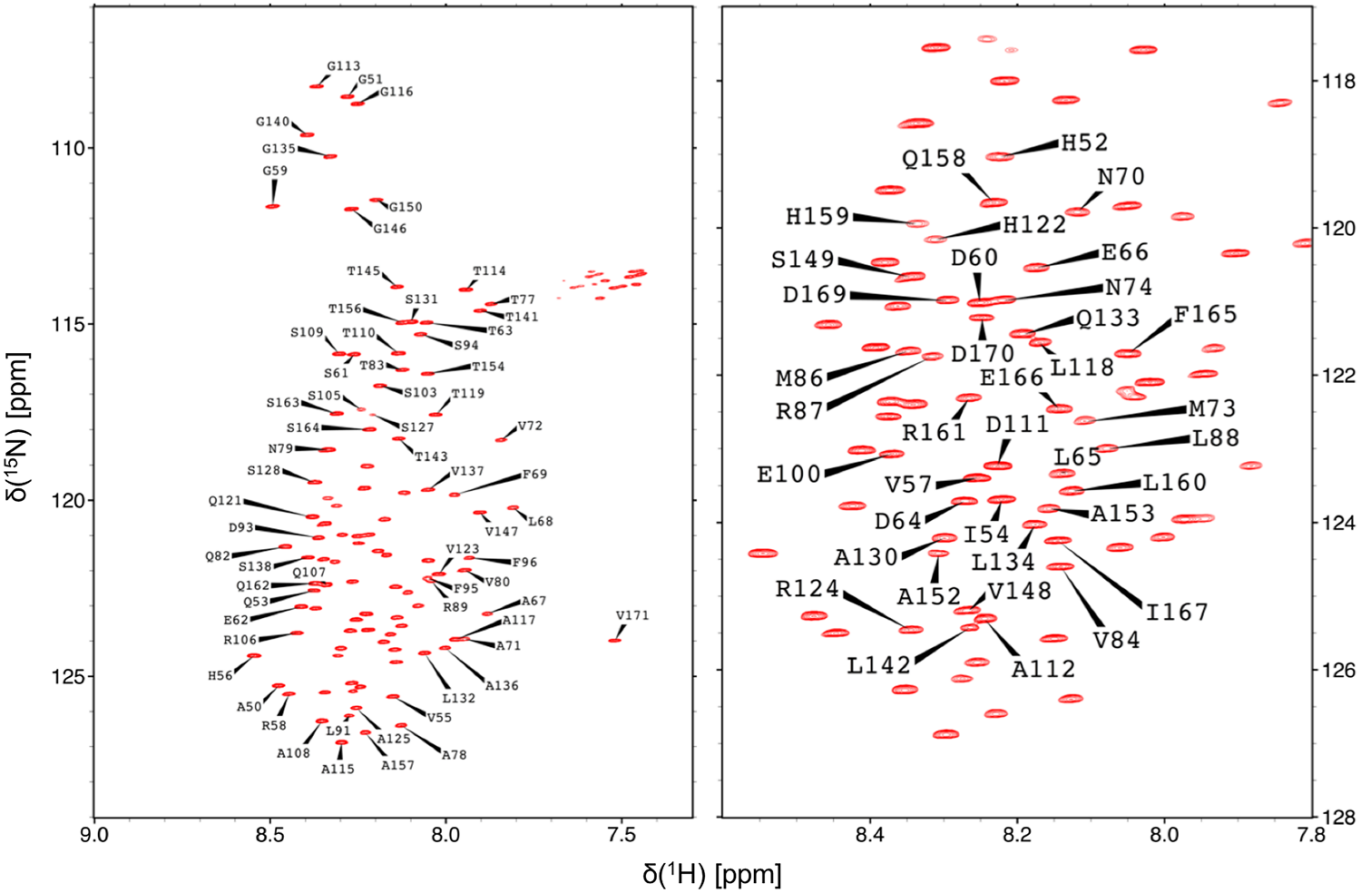
Left ^1^H–^15^N TROSY HSQC spectrum of YAP 50–171 at pH 6 and 298 K. Right magnification of the central region of the spectrum

**Fig. 2:**
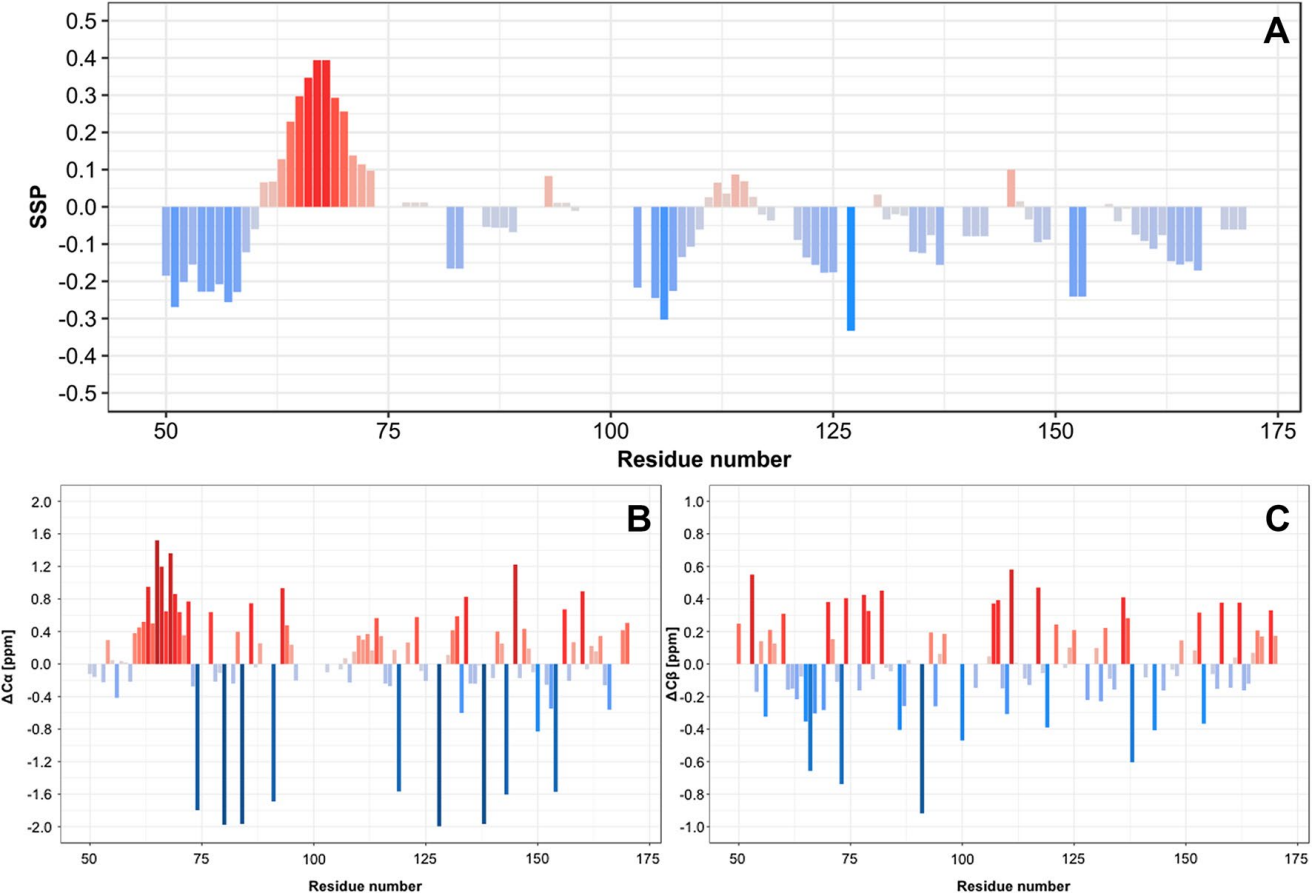
**a** SSP score (Marsh et al. 2006) of YAP 50–171 at pH 6 and 298 K. Positive scores indicate a propensity for α-helical structures, whereas β-strands or extended structural elements possess a negative score. The calculation of the SSP score was performed with all available chemical shift data. **b** Cα chemical shift deviations from random coil values (Zhang et al. 2003). **c** Cβ chemical shift deviations from random coil values

Assignment of the backbone amides ^1^H, ^15^N of YAP 50–171 is shown in Fig. 1. Backbone amide resonances were assigned for all of 108 non-proline residues, except of K76, K90, K97, K102, H104, and H126. Therefore, 12% of the total ^1^H resonances of the protein were assigned. In addition, 94% of Cα and Cβ resonances of the respective residues and 81% of C’ resonances were assigned. The very narrow peak dispersion in the ^1^H dimension of the ^1^H–^15^N HSQC spectrum shows that YAP 50–171 is intrinsically disordered. Nevertheless, the secondary structure propensity (SSP) score (Marsh et al. 2006) clearly indicates a propensity for a preformation of the α-helix and the N-terminal β-strand in the unbound state (Fig. 2a). The protein region of the Ω-loop shows no exceptional SSP scores. Furthermore, the protein seems to preferentially adopt extended structures (negative SSP scores).

The ^1^H, ^13^C and ^15^N chemical shifts have been deposited in the BioMagResBank (http://www.bmrb.wisc.edu/) under the BMRB accession number 27290.
